# Establishing a novel community-focussed lactation support service: a descriptive case series

**DOI:** 10.1186/s13006-021-00446-5

**Published:** 2022-01-15

**Authors:** Samantha Griffin, Jo Watt, Sophie Wedekind, Solange Bramer, Yasmin Hazemi-Jebelli, Robert Boyle, Gillian Weaver, Natalie S. Shenker

**Affiliations:** 1 Department of Surgery and Cancer, Imperial College London, England, W12 0HS UK; 2 The Human Milk Foundation, Rothamsted Institute, Hertfordshire, AL5 2JQ UK; 3 Imperial College London Medical School, St Mary’s Hospital, London, W2 1NY UK; 4 Department of Paediatrics, Imperial College London, St Mary’s Hospital, London, W2 1NY UK

**Keywords:** Donor human milk, Supplementation, Breast cancer, Surrogacy, Perinatal mental health, Milk bank, Safety, Infant feeding

## Abstract

**Background:**

Although breastfeeding is widely acknowledged as protecting both infant and maternal health postnatally, a partial or complete shortfall of maternal milk can occur for a range of reasons. In this eventuality, the currently available options for feeding infants are screened donor human milk (DHM), infant formula or unscreened shared human milk. In the UK, DHM has only been widely available in specific clinical contexts for the last 40 years, mainly to reduce the risk of necrotising enterocolitis in extremely preterm infants alongside optimal support for maternal lactation and breastfeeding. The Hearts Milk Bank (HMB) was established in 2017 as an independent, non-profit human milk bank that aimed to ensure equitable, assured access to screened DHM for neonatal units. As a result of the generosity of mothers, a surplus of DHM rapidly became available and together with lactation support, has since been provided to families with a healthcare referral. This programme has now been formalised for families facing lactational challenges, and DHM stocks are permanently maintained to meet their needs.

**Case series:**

This case series describes the clinical paths of four families who accessed lactation support and DHM from the HMB, along with a description of the process for community provision. To date, the HMB has supported over 300 families. Working collaboratively with key stakeholders, the HMB team has developed a prioritisation strategy based on utilitarian ethical models, protocols that ensure safe handling and appropriateness of use, broader donor recruitment parameters that maintain safety with a pragmatic approach for full term healthy infants, and a process to ensure parents or carers have access to the knowledge needed to give informed consent and use DHM appropriately.

**Conclusions:**

Stakeholders, including parents, healthcare professionals, and milk banks, will need to discuss priorities for both DHM use and research gaps that can underpin the equitable expansion of services, in partnership with National Health Service (NHS) teams and third-sector organisations that support breastfeeding and maternal mental health.

## Background

Donor human milk (DHM) is expressed by screened lactating women who have milk surplus to their own infant’s needs and freely given to a human milk bank, where it is processed and microbiologically tested. After the closure of most milk banks in the UK in the 1980s, DHM became highly rationed for only the most vulnerable premature infants cared for in neonatal intensive care units (NICUs), if used at all. This occurred despite recommendations from the World Health Organization (WHO) for DHM to be the first-line feed of choice for very low birth weight infants in the absence of maternal milk [1]. The most recent estimates show fewer than 30% of preterm infants have access to DHM in the UK [2]. The capacity of human milk banks globally is increasing, with over 700 active milk banks now operational, but UK provision remains NICU-focussed and fragmented [3]. In England, most milk banks are hospital-based, producing DHM for their own local neonatal unit(s) with some larger regional milk banks providing milk to neonatal units over a wider geographical region [4]. Scotland has a centrally commissioned national service, and Northern Ireland has a single milk bank that provides DHM across the whole island of Ireland, while Wales does not have a milk bank.

DHM is currently rarely funded by the National Health Service (NHS) outside of a NICU, and to gain funding families previously have had to apply to Clinical Commissioning Groups (CCG) with the support of their general practitioner. Over the last three years a novel community-focussed service has developed at the Hearts Milk Bank (HMB), supported by charitable funding through the Human Milk Foundation (HMF). In this model, the HMB supplies DHM and lactation support to families where NHS funding is not available, aiming to create evidence for future service development.

This case series examines the history of this service, describing four representative cases that illustrate the wide-ranging indications for DHM use and impacts, and the process that has developed to support the principle of utilitarian provision when DHM is prioritised according to greatest need.

## Community support by the hearts Milk Bank

In 2017, the Hearts Milk Bank was founded as the first independent, non-profit human milk bank in the UK, aiming to provide equitable, assured access to DHM for hospitals without a milk bank. The large number of women applying to become milk donors meant that from mid-2017 the HMB always had a surplus of milk available beyond the needs of hospitals.

In September 2017, after approaches by several families seeking DHM to support late- and full-term infants at home, the cofounders developed an informal service that provided DHM free-at-the-point-of-need following clinical request. In this manner, surplus DHM would not be wasted after the effort of milk donors and the milk bank team. A full medical history and feeding assessment by an HMB International Board-Certified Lactation Consultant (IBCLC) was carried out with each mother to ensure that an optimal plan was in place, and that the use of DHM would not undermine the chances of a mother establishing a full milk supply, where the family were breastfeeding their infant. Informed consent regarding how DHM is sourced, processed and used was obtained with a documented healthcare practitioner referral. Over time, awareness of this service spread through the healthcare community and demand increased (Table 1).

**Table 1:** Provision of donor human milk to families in the community from the Hearts Milk Bank within the first three years, with projected estimates for 2021 based on an extrapolation of the first six months

Year	No. recipient families	Total volume of milk provided (litres)
2018	5	499
2019	23	905
2020	64	1660
2021 (projected)	> 250	3000–5000 (estimated)

**Fig. 1:**
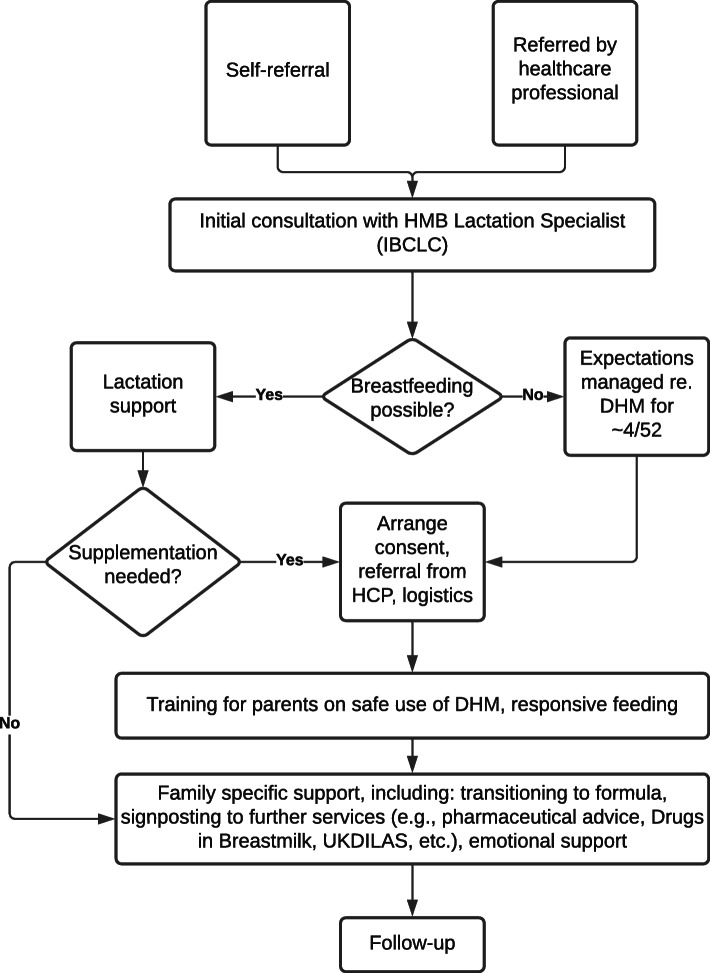
Process map for the provision of support to a family in the community. Abbreviations: *DHM* donor human milk; *HCP* health care provider; *HMB* Hearts Milk Bank; *IBCLC* International Board Certified Lactation Consultant; *UKDILAS* UK Drugs in Lactation Advisory Service

**Fig. 2:**
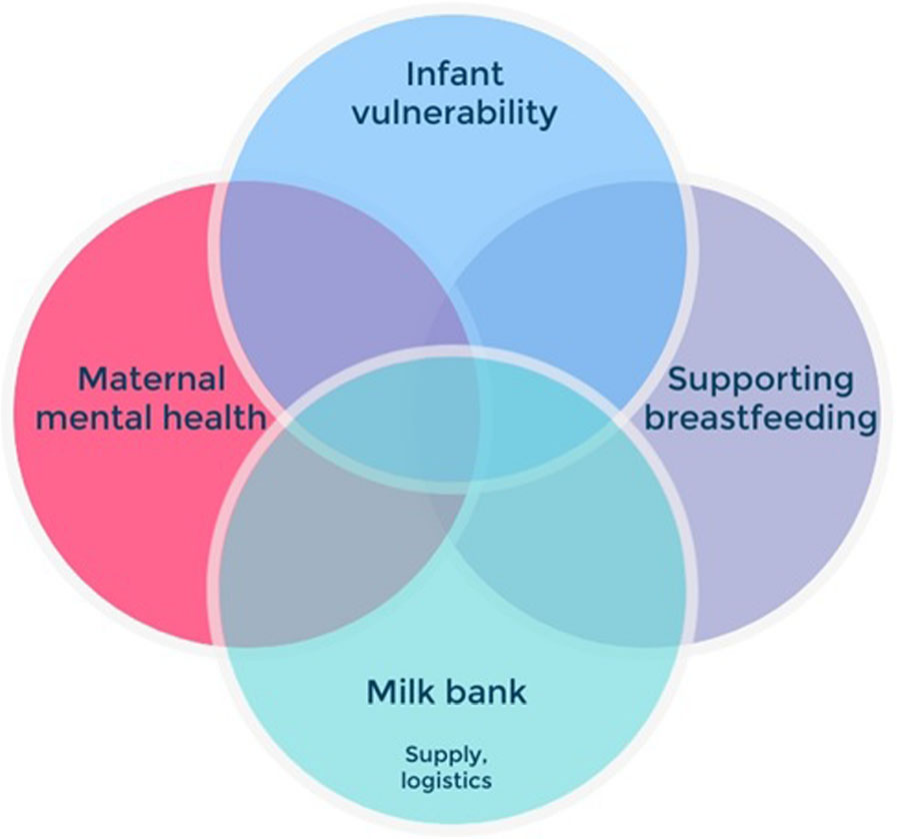
When considering DHM allocation: Infant vulnerability is prioritised, with support for maternal mental health and lactation treated equally, underpinned by the logistical difficulties and stock in the milk bank. Abbreviations: *DHM* donor human milk

As the service has grown as part of the Human Milk Foundation, the sophistication of community provision has increased and the capacity to meet the need has been funded by charitable activity so that the service continues to be free to parents. The HMF Prioritisation Panel was formed in 2018 to bring together stakeholders, organisations and individuals, to develop guidelines for the use and prioritisation of DHM outside of a hospital (Figs. 1 and 2).

The following case series describes the clinical scenarios of four families who have used this novel community service, followed by a description of the safeguards in place to support parental choice and protect infants and mothers by the utilisation of the prioritisation panel guidelines, and plans for future research. Interviews were conducted by telephone with one of the authors. Participants were informed about the case series and gave verbal consent to be included. They were then invited to talk about their experience, with the author asking questions if clarification was required.

### Case 1

Case 1 was a 33-year-old woman, gravida 2 para 0 (G2P0). After an uncomplicated pregnancy she was induced at 41^+ 1^ weeks gestation for suspected spontaneous rupture of membranes (SROM). After an emergency Caesarian section, her son (K) was born in good condition but deteriorated and required NICU admission at three hours. K was diagnosed with persistent pulmonary hypertension and required intubation and multiple courses of antibiotics. The mother experienced a post-epidural puncture causing significant pain which needed surgical correction. The mother reported feeling psychological trauma due to separation and K’s uncertain medical condition. She began pumping three-hourly to establish a milk supply but found initiating breastfeeding on the NICU challenging. K received her expressed breastmilk (EBM) and then formula via nasogastric tube (NGT).

After three weeks in the NICU, K was discharged home. The mother reported finding this very stressful and feeding continued to be difficult. The mother used a supplemental nursing system and continued to express three-hourly. Post-discharge K’s weight fell below the 0.05th centile. On the advice of local breastfeeding support groups, she started to top up with formula. Shortly after, K began to experience seizures and was readmitted to the NICU. He was diagnosed with bilateral perisylvian polymicrogyria (1p36 deletion syndrome), a rare neurological disorder affecting 1 in 5000 newborns that affects cerebral cortex folding, speech and causes swallowing difficulties.

Supplemental feeds had to be given via NGT as bottle feeding resulted in silent aspiration, but he had a safe swallow during breastfeeding. K remained in the hospital for another three weeks before going home on anti-epileptic medication under the care of a multidisciplinary team. During this admission, the mother requested domperidone to aid her milk supply. Although the initial clinical plan was to breastfeed three-hourly with simultaneous formula feeds via NGT and express milk in between, the mother found this regimen “unsustainable”. She self-referred to the HMB with the support of the hospital dietician when she was on the point of ending breastfeeding but desperate to continue as she had been advised by clinicians that it was the “single best thing to keep him well”, minimizing the risk of immunocompromise and seizure activity.

DHM was well-tolerated via a supplemental nursing system, and support from the HMB IBCLC enabled her to establish full breastfeeding, weaning off DHM supplementation within eight weeks; in total, the HMB supplied K with 16 L of DHM. K gained weight rapidly after DHM top-ups were implemented and developed better oral motor skills, enabling NGT removal. Around six months, he started to eat solid foods, and continued to develop well with a minor developmental delay. K attends nursery and continues to breastfeed at two-years-old.

The mother described struggling with the trauma she experienced related to K’s initial sepsis, seizures and diagnosis. She reported that being able to establish breastfeeding enabled her to establish a relationship with K and supported her healing, stating, “It allowed the pieces of me that were torn apart to be stitched back together”. She also felt immense pride and happiness in being able to donate surplus milk to the HMB as her son continued to grow.

### Case 2

Case 2 was a 34-year-old woman, G3P1. The mother had a three-year-old son who she had initially breastfed plus infant formula top-ups. He developed eczema at four months and additionally, several food allergies. In her next pregnancy, she was diagnosed with chronic myeloid leukaemia at 16 weeks gestation. The pregnancy continued with support from the haematology team. The mother reported breastfeeding was an immediate concern after diagnosis, driven by her desire to minimise the risk to her baby. She was told at 20 weeks she would not be able to breastfeed her baby as a result of incompatibility with treatment for leukaemia. DHM was suggested but her applications were rejected by multiple milk banks as being ‘outside their scope of practice’. She was unwilling to go through a CCG request as a result of her circumstances. She contacted the HMB and was able to arrange DHM at the first phone call.

At 30 weeks’ gestation, the mother’s white cell count continued to rise, and she required leukapheresis once or twice a week. Further intervention was avoided until she delivered following an induction of labour at 37 weeks. Her treatment commenced a few days after she gave birth, which enabled her to breastfeed initially before moving on to use DHM. She continued to use DHM for five weeks, receiving a total of 28 L from the HMB and then transitioned onto formula milk.

### Case 3

Case 3 was a 35-year-old G3P1 woman who had experienced a difficult breastfeeding journey with her first child. The mother reported notable tongue and lip ties coupled with Raynaud’s phenomenon and nipple vasospasm that resulted in “excruciating pain” on breastfeeding, but after tongue- and lip-tie division at ten weeks and maternal nifedipine, she weaned off the use of formula and breastfed until her first child was three and a half years old.

The mother became pregnant again with twins, born at 37 weeks’ gestation; after a normal vaginal delivery of twin one (a boy), the second twin (a girl) developed fetal distress. An emergency Caesarian section was performed under general anaesthetic and the mother experienced a massive obstetric haemorrhage with a total blood loss of 5000 mL. The mother required multiple red cell and platelet transfusions. After a difficult post-delivery recovery, including Intensive Therapy Unit and High Dependency Unit admissions, she was discharged but readmitted the next day with bilateral pneumonia. She subsequently developed empyema and a serious uterine infection requiring emergency surgery. During this period, the mother continued to attempt to pump to maintain her milk supply but became too unwell to continue.

When the twins were seven and a half weeks old, the mother attempted to reestablish lactation. The twins had been predominantly cared for by her husband and family and were both formula fed. Her clinical team advised her to stop re-lactating so she could commence oestrogen hormone replacement therapy for three months. This left the mother feeling devastated and guilty that she provided breastmilk for her first child and was unable to provide the same for her twins.

The mother self-referred to the HMB with the support of her general practitioner, and although her requirement fell outside of standard guidelines set at the time (the twins were three and a half months old), the team felt that providing enough DHM for one to two feeds each day for each twin would have a beneficial impact on the mother’s psychological health. The mother reported that after 48 h, her son’s eczema had improved and both twins had reduced constipation over the next few weeks. The HMB continued to provide milk and lactation support, and by six months postnatally she managed to fully re-lactate; the HMB supplied a total of eight litres DHM. The mother described the service as “more than milk”, and that re-lactating helped her overcome her postnatal trauma. The mother went on to donate milk to the HMB from her own surplus supply.

### Case 4

A 35-year-old G4P0 was informed about the HMB by her health care provider (HCP). She was HIV-positive, on antiretroviral treatment with an undetectable viral load during pregnancy, was planning a vaginal birth and wanted to breastfeed. She was being cared for by a specialist health advisor for reproductive health and wellbeing. The healthcare provider had discussed with her the option of receiving DHM as a backup during the early days of her breastfeeding journey, contacted the milk bank on her behalf and then provided the milk bank’s contact details to enable her to self-refer.

The current British HIV Association guidelines aim to minimise the risk of transmission through breastmilk by advising mothers to follow ‘The Safer Triangle’: only breastfeed if the viral load remains undetectable, both mother and baby are free of gastrointestinal problems, and the mother’s nipples remain healthy and without signs of infection [5]*.* If formula milk is introduced at any point, the mother is advised to only give formula milk from that point onward and to not mix feeds.

The mother had a straightforward pregnancy but required an induction of labour as she was overdue and went on to have an emergency Caesarian section for failure to progress. She could not achieve a good latch because of severely inverted nipples, but with DHM used sparingly as a supplement, fed her baby EBM without additional requirement for formula for the first four months of his life. The HMB supplied her with a total of eight litres DHM. She then transitioned onto formula without any adverse effects.

### HMB processes

#### Parent counselling

Prioritisation at the HMF is based on the ethical principles of equity and facilitating parental choice. It is based on four areas outlined in Fig. 2. The counselling stage is therefore essential for setting parameters for parental expectation**.**

In terms of counselling parents, further work will describe the details of the approach developed at the HMB. In brief though, it is important that sufficient time is given for each recipient caregiver to understand enough about how DHM is processed to make an informed decision. Although families can self-refer to this service, a clinical referral is also needed for each case to maintain safety and oversight of each dyad, and the recipient infants should be monitored by their HCP team as normal with regards to weight gain and development. Infants receiving DHM as sole nutrition should also receive standard multivitamins from two to three weeks postnatally, as some of the vitamin content of DHM is affected by processing, particularly vitamin C and some of the B vitamins [6]. DHM should be provided with minimal logistic barriers for parents, particularly for mothers experiencing trauma, including those diagnosed with cancer, to reduce additional stress. In particular, given 44 out of 44 CCG applications in the first year of the HMB operation were rejected, we no longer request parents to make extraordinary funding applications. The psychological distress caused by rejection for CCG funding cannot be underestimated, particularly for mothers who are unwell and deeply wish to breastfeed.

#### HMF prioritisation panel

In May 2019, the Human Milk Foundation established a panel of volunteer experts to achieve a consensus on how access to donor milk should be prioritised (Fig. 2). The panel includes a neonatologist, paediatrician, parents (donors and recipients), lactation specialists, milk bank team members and psychiatrist and psychologist. Initially, the panel met every six months, and now meets yearly to review the operation of the charity over the year and adjust guidelines over time. After discussion, donor milk is prioritised according to four parameters: infant vulnerability (hence NICU milk is always prioritised), maternal breastfeeding, maternal psychological health, and milk bank supply/logistics. Sometimes these overlap but they give the HMB lactation and logistics teams a broad context to manage a finite resource and set realistic expectations (Table 2).

**Table 2:** In terms of duration of donor milk supply, the Human Milk Foundation Prioritisation Panel set the following guidance

Indication	Examples	Guideline
Breastfeeding is impossible	Maternal cancer, maternal death, contraindicated medication, surrogacy and adoption where induced lactation is not possible.	Sufficient DHM to be offered for 4 weeks, followed by a taper period where formula is introduced gradually over 2–3 weeks
Milk supply issue	Post-partum haemorrhage, Sheehan’s syndrome, insufficient glandular tissue, breast hypoplasia, polycystic ovarian syndrome, gestational diabetes	No definitive cut-off. The aim is to provide DHM during the window of opportunity to maximise maternal milk supply, while offering optimal lactation and emotional support.

*DHM* donor human milk

The key to these discussions was the utilitarian ethical approach to provide support to as many families as possible, being transparent in what is possible so that caregivers’ expectations can be managed from the outset, and a structure that is simple and fair for the team to follow.

In the future, the HMF Prioritisation Panel aims to review how DHM capacity can be increased in the UK through a variety of approaches, including reassessing microbiological guidelines for milk failure, increasing the potential pool of donors through communications, pharmacological studies to assess the safety of different medications and assessing the viability of informal rules on polypharmacy of milk donors. Further work for the Prioritisation Panel is to ensure equity of this resource and service and set research priorities that can evaluate and extend the available services, including a formal modelling of DHM demand and donor recruitment potential, and understanding the impacts on infants of longer-term DHM use as the sole or partial source of nutrition.

## Discussion

The WHO recommends exclusive breastfeeding for the first six months of life followed by the introduction of complementary solid foods alongside continued breastfeeding until the age of two or beyond [7]. Despite current guidance from the National Institute for Health and Care Excellence (NICE) to provide information and support about breastfeeding to pregnant women and new families [8], the UK has some of the lowest breastfeeding rates in the world. The last UK infant feeding survey in 2010 identified that while there was an 81% initiation rate, only 46% of mothers were exclusively breastfeeding at one week and just 1% breastfed exclusively at six months [9]. There are common themes that give reasons to why a mother may stop breastfeeding earlier than planned: lactation factors such as a perceived low milk supply or positioning and attachment concerns, and maternal factors such as medicinal needs and psychosocial, and lifestyle factors such as wanting to share parental responsibilities of feeding [10–12]. Given few women (estimates of 1–2%) have complete primary milk insufficiency, tailored lactation support needs to be available to support women to overcome feeding difficulties and improve breastfeeding rates [12, 13]. DHM may play an important role in supporting maternal breastfeeding, as evidenced from recent observational studies published from India [14] and the USA [15, 16]. The sole published randomised controlled trial, which was conducted on a postnatal unit in the USA, demonstrated no increase in breastfeeding rates on discharge [17]. However, this study was confounded by the high rates of DHM use on the postnatal ward and consequent low uptake into the trial (only 60 entrants out of birth populations of several thousands over the study period), given that DHM supplementation was gold standard for that unit.

The United Nations has stated that breastfeeding is a human rights issue for both the mother and the child [18], but options are limited for women unable to breastfeed in the short- or long-term. The WHO recommends that infants who cannot receive their mother’s own milk or who need additional supplementation, should be fed DHM [1, 19]. There are no global guidelines on how to prioritise DHM. Despite global variation in use, and historic precedent for donor milk being used more widely in hospitals prior to the HIV pandemic, DHM has been recommended only for very low birth weight infants [1]. Such recommendations appear based on the perception that there would not be sufficient supply to meet the number and dietary needs of infants with higher weights and full-term infants. However, countries that have invested in milk bank services, such as Brazil, Canada and Norway, are able to support a much broader provision, including non-hospitalised infants [20]. As the smallest, most vulnerable pre-term infants are prioritised, limited research has evaluated the use of DHM outside of the NICU [21, 22]. Recent work by our group has suggested normal rates of growth in full-term infants fed solely or partially with DHM [23], with unexpected positive impacts on maternal mental health and wellbeing that will be further investigated in prospective studies (Brown et al. In Press).

A scoping review of the literature by McCune and Perrin identified 26 studies which utilised DHM in populations other than hospitalised pre-term infants [22]. They categorised these studies into five groups based on the type of recipient: adult, child, infants born after 35 weeks’ gestation with health risks, healthy infants born after 35 weeks’ gestation and post-discharge preterm infants. A service evaluation of UK milk banks in 2019 found that few NHS milk banks provided DHM to infants outside of the NICU, including mothers entirely unable to breastfeed, but on average the provision was less than one litre (unpublished audit results).

Despite a development in the understanding of the benefits of DHM use, there has been a lack of funding and infrastructure to support milk banking in the UK. Where investment has been made, notably in Brazil and North America, and where there is a policy and support from healthcare authorities towards milk banking, use of DHM has grown [24]. Brazil has over 219 milk banks with outreach in terms of training and support to over 23 countries, mostly in the low- and middle-income countries. In 2003 more than 100,000 preterm infants received donor milk in Brazil, and by 2015 at least 170,000 neonates received DHM and two million women received lactation support [25]. Breastfeeding rates in Brazil have risen in the last 20 years from 4 to 76% at 6 months, contributing to a halving of child mortality [26].

From a global perspective, DHM is available in the community setting in several countries. Tully et al. (2004) described three cases in the USA where DHM was used successfully for three children with complex neurological conditions [27]. The four non-profit milk banks in Canada each prioritise recipients using a triage list, first to inpatients and then to community infants, where supplies allow [28]. In China two reviews have shown over 94% DHM recipients were premature infants, but DHM was used to feed infants with conditions including cystic fibrosis, failure to thrive, congenital anomalies, necrotising enterocolitis, immune deficiencies, postoperative therapy in infants with cardiac anomalies, cancer, feed intolerances, severe infection, malnutrition after major surgery, or other situations including lack of breastfeeding because of mother’s illness, as well as adult pathologies [29]. In Norway, it has been noted that families were able to access DHM on a few occasions and for a fee, but that community babies are not a prioritised group [30]. In India, “Lactation Management Centres” have been developed to provide a dedicated space within health facilities, not only to provide lactation support, but to provide a central place for the donation, processing and distribution of DHM [31]. Given this limited access to DHM in the community, it is unsurprising that informal sharing of milk, whereby breast milk is obtained from a mother other than the infant’s own without screening and processing from a milk bank, is burgeoning [32].

## Conclusions

While preterm and sick infants must always be prioritised, the provision of DHM should no longer be thought the exclusive preserve of the NICU. Further research is urgently needed to clarify the impacts of DHM availability on maternal mental health and breastfeeding support, in addition to driving innovation in this sector while maintaining the highest standards of ethics and equity in the considering prioritisation of what will continue to be a limited resource in the near future. Further developing collaborations between academics, local authorities and other children’s services in both the public and third-sector will lay the foundations for a truly equitable service for all families who could benefit.

## Abbreviations

CCG: Clinical commissioning group
DHM: Donor human milk
HCP: Health Care Provider
HMB: Hearts Milk Bank
HMF: Human Milk Foundation
IBCLC: International Board-Certified Lactation Consultant
NHS: National Health Service
NICU: Neonatal intensive care unit
WHO: World Health Organization
